# Red blood cell transfusion-related eicosanoid profiles in intensive care patients—A prospective, observational feasibility study

**DOI:** 10.3389/fphys.2023.1164926

**Published:** 2023-03-16

**Authors:** Pierre Raeven, Gerhard Hagn, Laura Niederstaetter, Jonas Brugger, Sophia Bayer-Blauensteiner, Christoph Domenig, Konrad Hoetzenecker, Martin Posch, Gerda Leitner, Christopher Gerner, David M. Baron

**Affiliations:** ^1^ Division of General Anesthesia and Intensive Care, Department of Anesthesia, General Intensive Care and Pain Management, Medical University of Vienna, Vienna, Austria; ^2^ Department of Analytical Chemistry, Faculty of Chemistry, University of Vienna, Vienna, Austria; ^3^ Center for Medical Statistics, Informatics and Intelligent Systems, Section for Medical Statistics, Medical University of Vienna, Vienna, Austria; ^4^ Division of Vascular Surgery, Department of Surgery, Medical University of Vienna, Vienna, Austria; ^5^ Department of Thoracic Surgery, Medical University of Vienna, Vienna, Austria; ^6^ Department of Blood Group Serology and Transfusion Medicine, Medical University of Vienna, Vienna, Austria; ^7^ Joint Metabolome Facility, Faculty of Chemistry, University of Vienna, Vienna, Austria

**Keywords:** transfusion-related immunomodulation, storage lesion, transfusion, eicosanoid, arachidonic acid, hydroxylated eicosatetraenoic acid

## Abstract

**Introduction:** Eicosanoids are bioactive lipids present in packed red blood cells (PRBCs), and might play a role in transfusion-related immunomodulation (TRIM). We tested the feasibility of analyzing eicosanoid profiles in PRBC supernatant and in plasma samples of postoperative intensive care unit (ICU) patients transfused with one unit of PRBCs.

**Methods:** We conducted a prospective, observational feasibility study enrolling postoperative ICU patients: 1) patients treated with acetylsalicylic acid following abdominal aortic surgery (Aorta); 2) patients on immunosuppressants after bilateral lung transplantation (LuTx); and 3) patients undergoing other types of major surgery (Comparison). Abundances of arachidonic acid (AA) and seven pre-defined eicosanoids were assessed by liquid chromatography and tandem mass spectrometry. PRBC supernatant was sampled directly from the unit immediately prior to transfusion. Spearman’s correlations between eicosanoid abundance in PRBCs and storage duration were assessed. Patient plasma was collected at 30-min intervals: Three times each before and after transfusion. To investigate temporal changes in eicosanoid abundances, we fitted linear mixed models.

**Results:** Of 128 patients screened, 21 were included in the final analysis (Aorta *n* = 4, LuTx *n* = 8, Comparison *n* = 9). In total, 21 PRBC and 125 plasma samples were analyzed. Except for 20-hydroxyeicosatetraenoic acid (HETE), all analyzed eicosanoids were detectable in PRBCs, and their abundance positively correlated with storage duration of PRBCs. While 5-HETE, 12-HETE/8-HETE, 15-HETE, 20-HETE, and AA were detectable in virtually all plasma samples, 9-HETE and 11-HETE were detectable in only 57% and 23% of plasma samples, respectively.

**Conclusions:** Recruitment of ICU patients into this transfusion study was challenging but feasible. Eicosanoid abundances increased in PRBC supernatants during storage. In plasma of ICU patients, eicosanoid abundances were ubiquitously detectable and showed limited fluctuations over time prior to transfusion. Taken together, larger clinical studies seem warranted and feasible to further investigate the role of PRBC-derived eicosanoids in TRIM.

## Introduction

Transfusion of packed red blood cells (PRBCs) has been associated with increased morbidity and mortality in patients admitted to the intensive care unit (ICU) (Marik and Corwin, 2008; Rohde et al., 2014; Flatman et al., 2021; Flatman et al., 2022). One possible factor involved in these altered outcomes is transfusion-related immunomodulation (TRIM), which includes immunosuppressive and proinflammatory effects induced by PRBC transfusion (Remy et al., 2018; Yuan et al., 2020; Xu et al., 2021). Detailed mechanisms of this poorly defined phenomenon are largely unknown, but accumulation of immunomodulators in the PRBCs as part of the so-called “storage lesion” may play a role (Claridge et al., 2002; Malone et al., 2003; Taylor et al., 2006). Eicosanoids are metabolic products of polyunsaturated fatty acids (PUFAs) such as arachidonic acid (AA). Prior studies have associated prolonged storage of PRBCs with an accumulation of AA and hydroxyeicosatetraenoic acids (HETEs) as its downstream products of oxidation (Silliman et al., 2011; Zimring et al., 2014; Fu et al., 2016; Peters et al., 2017; D'Alessandro et al., 2019). Eicosanoids may therefore be involved in the development of TRIM.

However, the non-routine analysis of eicosanoids precludes large retrospective studies to test their role *in vivo*. To date, plasma levels of eicosanoids following PRBC transfusion have only been studied in healthy volunteers receiving autologous blood after administration of lipopolysaccharide to mimic infection. To our knowledge, no prospective clinical study has addressed the underlying mechanisms of TRIM in the ICU in detail. This is not surprising, as it is difficult to predict PRBC transfusion in the ICU setting, impeding patient recruitment. Moreover, modern concepts of patient blood management reduce the frequency of PRBC transfusions (Leahy et al., 2017; Althoff et al., 2019), further impeding recruitment.

As patients admitted to the ICU postoperatively may be particularly susceptible to possible adverse effects of PRBC transfusion (Shankar Hari and Summers, 2018), a personalized transfusion strategy is desirable in these patients. Further understanding of eicosanoid dynamics during PRBC storage and after transfusion should help develop such individual transfusion strategies (D'Alessandro, 2019). In particular, PRBCs units could be matched with the immunological state and co-medication of each patient.

Thus far, eicosanoids have not been assessed in plasma of postoperative ICU patients receiving a PRBC transfusion. Therefore, the goal of this study was to provide feasibility data in order to facilitate the design of larger studies investigating the transfusion-related dynamics of eicosanoids and their immunomodulatory effects in a clinical setting. We hypothesized that 1) Assessment of eicosanoid abundance before and after PRBC transfusion would be feasible in a postoperative ICU setting, 2) Eicosanoid abundance in PRBCs would be detectable and would correlate with storage duration, 3) Eicosanoids could be detected in patient plasma and show stable baseline values, 4) Eicosanoid abundances in patient plasma would increase after transfusion.

## Materials and methods

### Ethics approval statement

All procedures performed were in accordance with the ethical standards of the institutional review board of the Medical University of Vienna (1595/2018) and with the 1964 Helsinki declaration and its later amendments. All patients included in the study provided written informed consent. The manuscript was drafted based on the STrengthening the Reporting of OBservational studies in Epidemiology (STROBE) checklist (Vandenbroucke et al., 2007).

### Study design and sample size estimation

Prior to this single-center feasibility study, there was no data available to estimate the effect size. We anticipated that not every screened patient would require a PRBC transfusion. Based on transfusion rates at our hospital, we estimated that we could recruit 10 patients per group within the pre-defined 2-year study period, resulting in a total of 30 patients.

To explore the effects of concurrent medication such as acetylsalicylic acid (ASA) and immunosuppressants on transfusion-related eicosanoid plasma abundances, we defined three groups: 1) patients treated with ASA admitted after aortic surgery (Aorta); 2) patients on immunosuppressants following bilateral lung transplantation (LuTx); and 3) patients admitted to the ICU after other types of surgery not treated with ASA or immunosuppressants (Comparison).

### Patient screening

Screening was scheduled from 1 December 2018 until 30 November 2020. We aimed to screen all patients scheduled for LuTx or open abdominal aortic surgery (bifemoral prosthesis, aortic replacement or aortic bypass surgery) as well as patients admitted to the ICU after major surgery. We included adult patients transfused with one unit of PRBCs after postoperative admission to the ICU.

As following drugs were expected to have confounding effects, their use was an exclusion criterion for specific groups in our study: Celecoxib, etoricoxib, parecoxib, ibuprofen, diclofenac, naproxen, and cysteinyl leukotriene receptor antagonists in all groups; ASA and protamine in the LuTx and Comparison groups; and glucocorticoids given within 24 h of transfusion in the Aorta and Comparison groups (Hobbhahn et al., 1991; Szefel et al., 2015). Further exclusion criteria were pregnancy and less than 12 h elapsed since the last PRBC transfusion.

### Blood sampling

**FIGURE 1:**
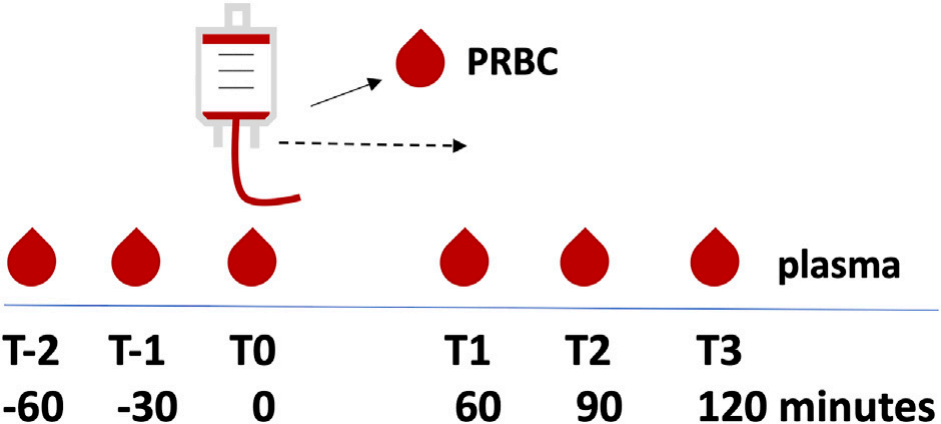
Overview of sampling time points. Plasma was sampled at 60, 30, and 0 min before transfusion (T-2, T-1, and T0, respectively), followed by transfusion of one unit of packed red blood cells (PRBC) over 1 h +/− 5 min. Plasma was then sampled at 60, 90, and 120 min after the start of the PRBC transfusion (T1, T2, and T3, respectively). In addition, one sample was drawn from the PRBC unit. The dashed line indicates the transfusion period.

Figure 1 illustrates the sampling schedule. One sample per patients was collected directly from the unit immediately prior to transfusion. Patient plasma was collected at 30-min intervals: Three times both before (to test baseline fluctuation in ICU patients) and three times after transfusion (to compare to baseline). Peripheral blood from patients was drawn from indwelling arterial catheters using ethylenediaminetetraacetic acid anticoagulation. Blood was collected at three time points to test the baseline fluctuation of eicosanoids in ICU patients (i.e., T-2, T-1, and T0 at 60, 30, and 0 min before transfusion, respectively). Then, one unit of PRBCs was transfused over 1 h +/− 5 min. After transfusion, blood was sampled at three additional time points (i.e., T1, T2, and T3 at 60, 90, and 120 min after the start of PRBC transfusion, respectively).

### Eicosanoid analysis

Eicosanoid abundance in PRBCs and patient plasma was analyzed by liquid chromatography and tandem mass spectrometry. A detailed description of the analytical methods is provided in the Supplementary Material. Eventually, eicosanoid abundances were expressed as normalized area under the curve (nAUC): AUC of the chromatographic peaks were divided by the mean AUC quantified for all standards of the corresponding sample. Traces with peaks that did not exceed a standardized arbitrary threshold were defined as under the lower limit of detection (LLD).

### Statistical analysis

In the whole cohort, Spearman’s correlations between eicosanoid abundance in PRBCs and storage duration were assessed. To descriptively test for fluctuations of eicosanoid abundance prior to transfusion, the relative differences of maximum and minimum values relative to the mean abundance of T-2, T-1, and T0 for a given patient and eicosanoid were calculated. Here, because not all analytes were detectable in all plasma samples prior to transfusion (T-2, T-1, T0), values under LLD were defined as half the minimum value of the specific eicosanoid in all measured samples.

To investigate whether eicosanoid abundances changed over time in each of the study groups, we fitted linear mixed models, which are described in detail in the Supplementary Material. Model estimates have to be interpreted as relative change compared to baseline values. The slope has to be interpreted as the relative change over 1 hour. Thus, values less then one would imply a decrease of the respective normalized abundance over time. Slopes of different study groups have to be interpreted as the relative changes over time within the group. The Comparison group was chosen as the reference category in every model when comparing contrasts between groups.

The significance level was chosen as *α* = 0.05. As no correction for multiple testing was applied, all *p*-values are of descriptive, hypothesis-generating character.

Statistical analysis was done using R, version 3.6.1 or higher (https://www.R-project.org/). Mixed models were computed using the package lmerTest (Kuznetsova AB and Christensen, 2017) and tobit regression models with the package Vector Generalized Linear and Additive Models (Yee, 2015). Graphs were plotted using GraphPad Prism 9.

## Results

### Patient recruitment

**FIGURE 2:**
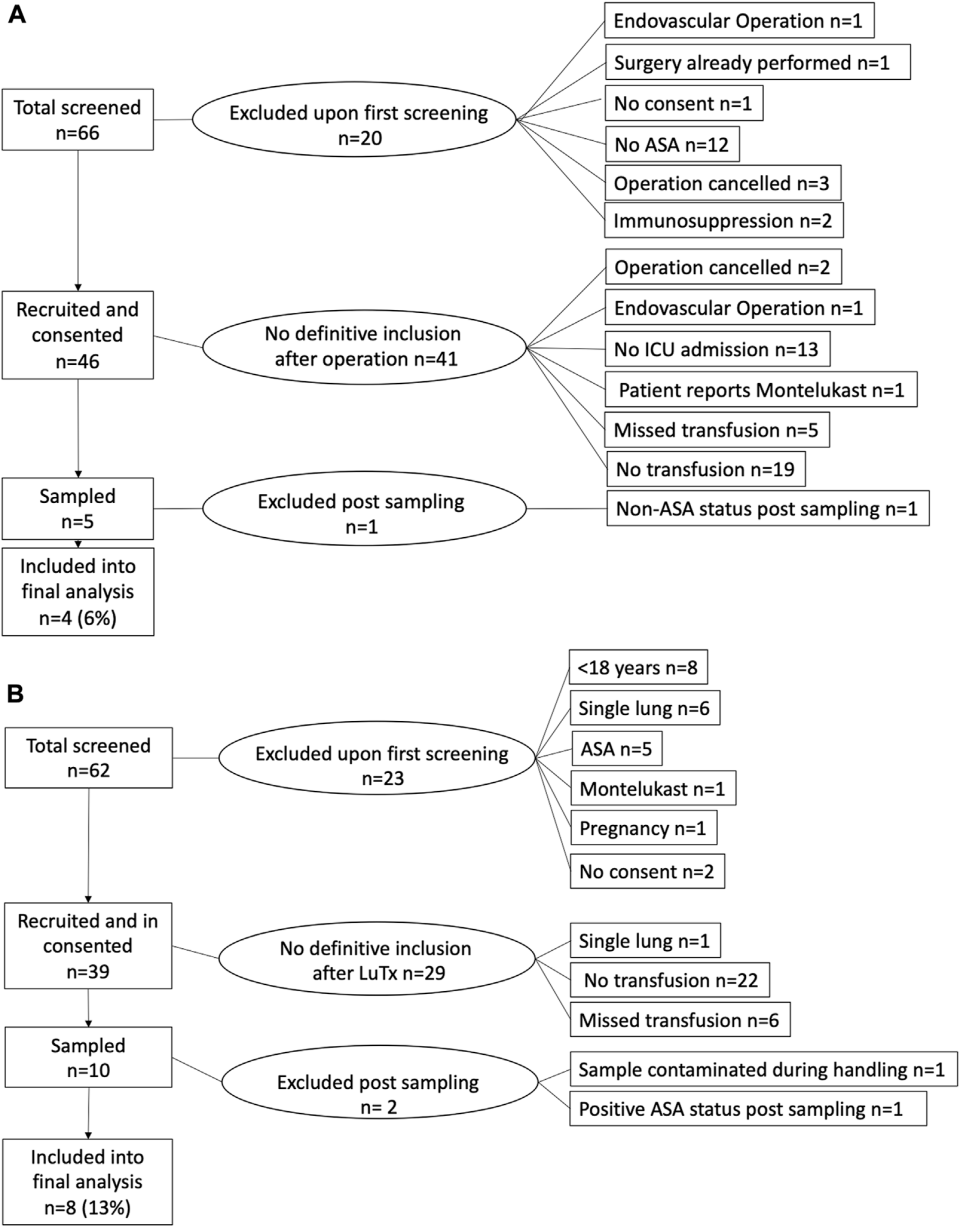
Flowcharts of patient recruitment. Patients scheduled for **(A)** aortic surgery or **(B)** bilateral lung transplantation were pre-screened, and informed consent was obtained before surgery. Definitive inclusion into the study was performed after surgery. In total, three patients had to be excluded after initial inclusion into the study. ASA, acetylsalicylic acid; ICU, intensive care unit; LuTx, bilateral lung transplantation.

Figure 2 shows the flow diagram of recruitment in the Aorta and LuTx groups. In total, 66 patients planned for aortic surgery (Figure 2A) and 62 patients planned for LuTx (Figure 2B) were screened preoperatively. Forty-six Aorta patients and 39 LuTx patients were eligible for inclusion and provided written informed consent. Of these, 10 Aorta patients and 16 LuTx patients required PRBC transfusion during their postoperative ICU stay. We were able to perform sampling in *n* = 5 (Aorta) and *n* = 10 (LuTx). In the remaining eligible patients, sampling was not performed because the sampling team was unavailable at the time point of transfusion or was not informed by the ICU team on call. Simultaneously, nine postoperative ICU patients (Comparison group) were recruited and sampled during the study period.

Samples of one patient in the LuTx group were contaminated during handling. Upon analysis, one included patient in the Aorta group had not received ASA, while one patient in the LuTx group had taken ASA. After exclusion of these two patients, 21 patients (Aorta *n* = 4, LuTx *n* = 8, Comparison *n* = 9) were included in the final analysis. Especially in the Aorta group, recruitment was slow, whereas recruitment in the LuTx group was ahead of schedule. Due to the Corona Virus Disease-19 pandemic, operative and ICU capacities were temporarily limited at our institution. Thus, recruitment was prematurely terminated on 19 March 2020 after 16 months, and the Ethics Committee was informed.

### Patient characteristics

Table 1 and Supplementary Table S1 show the demographic and clinical characteristics of patients included in the final analysis. Supplementary Table S2 shows co-existing diseases of the study population.

**TABLE 1:** Main patient characteristics.

Group	Pat ID	Age	Gender	Diagnosis	Operation	POD	PRBC storage duration (d)	PRBCs transfused during surgery (n)	Total PRBCs at ICU (n)
Aorta	34	65	m	Peripheral arterial vascular disease	Axillofemoral bypass	1	11	2	2
	36	77	m	Abdominal aortic aneurysm	Bifemoral prothesis	2	24	0	2
	48	82	f	Abdominal aortic aneurysm	Bi-iliacal prothesis	1	17	3	4
	81	81	m	Abdominal aortic aneurysm	Aortic replacement	2	29	1	4
LuTx	10	65	m	Fibrosis	LuTx	3	25	5	34
	12	32	f	Idiopathic pulmonary hypertension	LuTx	1	23	9	6
	14	59	f	Chronic obstructive pulmonary disease	LuTx	3	36	2	1
	15	38	m	Cystic fibrosis, transplant dysfunction	Re-LuTx	3	31	7	2
	25	25	f	Cystic fibrosis	LuTx	3	29	10	1
	49	48	m	Cystic fibrosis, transplant dysfunction	Re-LuTx	8	18	22	5
	64	37	m	Fibrosis	LuTx	6	23	17	5
	73	33	m	Cystic fibrosis	LuTx	3	20	5	2
Comparison	17	36	m	Abdominal wall seroma	Multiple	5	22	7	6
	20	84	m	Neoplasma vesicae	Cystectomy	3	25	0	1
	43	56	m	Pericardial effusion	Pericardial drainage	4	33	0	17
	46	77	m	Status post subhepatic hematoma	Wound management	1	29	0	6
	61	59	m	Thymoma	Thymectomy	44	18	13	20
	68	59	m	Renal cell carcinoma	Partial nephrectomy	1	17	3	5
	79	72	f	Stomach perforation	Gastrectomy	1	13	1	1
	80	62	m	Neoplasma vesicae	Cystectomy	1	25	0	2
	87	59	m	Peripheral arterial vascular disease	Thrombectomy	2	20	0	3

Pat ID, patient identification number; PRBC, packed red blood cell; POD, postoperative day at time point of transfusion; d, days; ICU, intensive care unit; LuTx, bilateral lung transplantation; Re-LuTx; repeated bilateral lung transplantation; m, male; f, female.

### Eicosanoid abundances in PRBCs and their correlation with storage time

Supplementary Table S3 contains eicosanoid abundance in PRBCs. 20-HETE was not detected in any of the analyzed PRBC samples, whereas 5-HETE was detected in all but one sample. All other eicosanoids were ubiquitously detected in all analyzed PRBC samples. The nAUC of eicosanoids in the PRBC supernatant positively correlated with PRBC storage duration (Spearman’s rho ranged from 0.47 for 11-HETE to 0.75 for 9-HETE, all *p* < 0.05, Table 2).

**TABLE 2:** Correlations between storage duration and normalized abundance of eicosanoids.

Eicosanoid	Spearman’s rho	*p*-Value
AA	0.49	0.025
5-HETE	0.70	<0.001
9-HETE	0.75	<0.001
11-HETE	0.47	0.034
12-HETE/8-HETE	0.52	0.015
15-HETE	0.59	0.005

All Spearman ´s rho. Since 20-HETE, was not detected in stored packed red blood cells, no correlation analysis could be performed. AA, arachidonic acid; HETE, hydroxyeicosatetraenoic acid.

### Eicosanoid abundances in plasma

Supplementary Table S3 contains eicosanoid abundances in plasma, and Supplementary Figure S1 shows individual dynamics of eicosanoid abundances in patient plasma at predefined time points for all patients, color-coded by group. One sample (T2 of patient #81) was contaminated during analysis, resulting in analysis of a total of 125 plasma samples. The eicosanoids AA, 5-HETE, 12-HETE/8-HETE and 20-HETE were detectable in all 125 plasma samples. In contrast, 15-HETE was detectable in all but one plasma sample, 9-HETE in 83/137 (61%) and 11-HETE in 36/137 (26%) of all available plasma samples.

### Fluctuations of eicosanoid abundance in pre-transfusion plasma

We hypothesized that normalized abundances of eicosanoids in plasma samples drawn at three time points within 90 min before PRBC transfusion (T-2, T-1, T0) would not vary outside the 0.5- to 2-fold range of their mean value. Fluctuation was indeed within this range in 95.2% of baseline measurements for AA, 11-HETE and 20-HETE, in 90.5% for 5-HETE and 15-HETE, but only in 76.2% and 61.9% for 12-HETE/8-HETE and 9-HETE, respectively.

### Estimated baseline eicosanoid abundances and dynamics over time

Despite the limited sample size, we performed an exploratory statistical analysis of eicosanoid abundances and dynamics before and after transfusion (Supplementary Table S4). Estimated eicosanoid abundances were not different between study groups at baseline. We estimated that every hour, 12-HETE/8-HETE and 20-HETE abundances decreased by 11% (slope 0.89, 95% CI 0.83–0.96) and 9% (slope 0.91, 95% CI 0.85–0.98) in the whole cohort (*p* < 0.01). Even larger decreases were seen in the Comparison group when this group was analyzed separately. In the isolated LuTx Group, only 20-HETE showed a significant (*p* < 0.01) hourly decrease by 15% (slope 0.85, 95% CI 0.77, 0.94). No significant differences between estimated slopes of eicosanoid normalized abundance in plasma could be found between either the Aorta group vs. the Comparison group or LuTx group vs. the Comparison group.

### Exploratory analysis of additional eicosanoids

In our setup, 47 additional analytes potentially involved in TRIM were identified (Supplementary Table S3). Sixteen of these analytes could be identified as known PUFAs or their isoforms, eighteen were known eicosanoids or isoforms, and thirteen were unknown. Two PUFAs and two PUFA isoforms, six known eicosanoids and one unknown analyte with an eicosanoid-specific m/z value indeed showed a correlation (r > 0.4 or r < −0.4, *p* < 0.05) between their abundance in the PRBC and PRBC storage duration, respectively (Supplementary Table S3). All analytes and their metabolic pathways (Steer et al., 2013; Gabbs et al., 2015) are shown in Supplementary Figure S2.

## Discussion

In this study, we intentionally used a small cohort to test whether analysis of eicosanoid dynamics associated with allogenic transfusion of PRBCs in postoperative ICU patients is feasible. We found that recruitment of ICU patients into this transfusion study was challenging, but feasible. Additionally, we were able to confirm that abundance of eicosanoids in PRBCs positively correlated with their storage duration. Eicosanoids were detectable in the majority of ICU patient plasma samples, and their abundances showed limited temporal fluctuation at baseline before PRBC transfusion.

Since timing of PRBC transfusion in the postoperative ICU setting is not predictable, one main focus of this study was the screening process itself. In another study evaluating the effects of PRBC transfusion on iron metabolism in ICU patients, only 2% of screened patients were included in the final analysis due to logistic problems (Boshuizen et al., 2019). Despite premature termination of our study due to the Corona Virus Disease-19 pandemic, we were able to include 9% of screened patients in the Aorta and LuTx groups in the final analysis. Our non-routine sampling procedures required immediate processing of samples, occupying at least one person during a 4-h period. Sampling was performed between 7 pm and 7 am and/or on Saturday/Sunday in 6 out of 21 patients. Study investigators were not informed or were unavailable for 11 eligible patients (5 in the Aorta and 6 in the LuTx group).

The bioanalytical results obtained in this study are in line with observations from previous studies, namely, accumulation of eicosanoids in stored PRBCs (Silliman et al., 2011; Zimring et al., 2014; Peters et al., 2017; D'Alessandro et al., 2019). Of note, previous studies have measured eicosanoid levels at 2 or 4 time points during storage (Silliman et al., 2011; Peters et al., 2017). In contrast, we assessed one sample per unit at the time point of transfusion. Despite the limited sample size (*n* = 21), we report a statistically significant positive correlation between normalized abundances of eicosanoid in stored PRBCs and PRBC storage duration. This study applied both a targeted and an untargeted approach. We identified AA and 7 predefined eicosanoids and another 47 PUFAs, eicosanoids, and features with eicosanoid-specific m/z values potentially involved in TRIM.

Especially in postoperative ICU patients, eicosanoid levels in plasma may vary considerably within short periods. To analyze fluctuations over time and to strengthen the robustness of baseline values prior to transfusion, we decided to collect a total of 3 baseline samples within 60 min. Most analytes showed fluctuation at baseline that were grossly within the hypothesized 0.5- to 2-fold range of their mean value.

Although not the main focus of this study, we performed a statistical analysis of eicosanoid dynamics before and after PRBC transfusion. However, the small cohort sizes and exploratory nature of the experiments require a cautious interpretation and statistics are of a descriptive, hypothesis-generating character. D'Alessandro et al. reported an increase in 9-HETE and 8-HETE in volunteers transfused with autologous PRBCs stored for 42 days (D'Alessandro et al., 2019). In contrast, transfusion of PRBCs did not result in the hypothesized increase of eicosanoids in plasma in our study. Abundances of 12-HETE/8-HETE decreased by 11% in the whole cohort and by 14% in the Comparison group. Similarly, nAUCs of 20-HETE (which was not detectable in stored PRBCs) decreased by 9% in the whole cohort and by 12% in the Comparison group. Furthermore, 20-HETE decreased by 15% in the LuTx group. These contrasting dynamics of eicosanoid abundances in plasma after transfusion may be explained by several differences between the two studies: mean storage duration of 23 days (minimum 11 days, maximum 36 days) in our study as compared to 42-day storage reported by D’Alessandro et al. (D'Alessandro et al., 2019), allogenic vs. autologous transfusion, postoperative ICU patients vs. healthy volunteers, intravenous transfusion of 250 mL of PRBCs over 1 h vs. intra-arterial transfusion of 138 mL of PRBCs within 9–12 min, sampling out of a radial arterial line at 60–120 min after start of transfusion vs. immediate venous sampling from the antecubital vein after intraarterial transfusion and acetylcholine infusion. In addition, we might have missed an immediate increase and subsequent decrease of eicosanoid abundances in plasma within the first 60 min after transfusion due to the sampling time points chosen. Short-lasting effects on normalized abundances of eicosanoids in plasma after PRBC transfusion may be quickly buffered by the patient’s blood, which is compatible with our biochemical understanding so far (Gerner et al., 2021). We did not observe any differences between estimated slopes of post-transfusion eicosanoid abundances in plasma among study groups. Although this may indicate that the underlying cause of ICU treatment and co-medication may not be major determinants of the effect of PRBC transfusion upon eicosanoid abundances in plasma, our study was most likely underpowered to investigate such an effect.

The low sample size was one of the limitations of our study. Although we have analyzed the data extensively, analysis was restricted by a relatively high number of measurements under LLD and thus heavily relied on model estimations. In addition, the large number of comparisons and the lack of correction for multiple testing necessitates cautious interpretation of these results. As our main focus was to test feasibility of the study design, no conclusions regarding the role of accumulating eicosanoids in TRIM can be made. Moreover, the lack of analytical calibrants and internal standards for several eicosanoids precluded determination of absolute eicosanoid concentrations, which have been reported in other studies (Silliman et al., 2011; Zimring et al., 2014; Fu et al., 2016; Peters et al., 2017). Our untargeted analysis approach, at first sight, hinders direct comparison between studies. However, this approach followed recent expert recommendations favoring the description of relative changes of nAUCs within studies, which may actually allow for a more robust comparison with other reports (Gladine et al., 2019). For larger future studies, possible clinical confounders should also be taken into consideration. Chronic disease, patients’ comorbidities, and concomitant medication may influence eicosanoid levels in plasma. Additionally, characteristics of the operation itself (e.g., duration, tissue damage, transfusion of blood products, extracorporeal circulation) may be a biasing factor. Postoperatively, varying critical conditions of ICU patients and various specifics of treatment in the ICU (e.g., nutrition and nutritional status, fluid resuscitation, extracorporeal therapies, variation in medication and route, duration and amount of transfusion of blood products) may play a role. Although we have collected data on multiple abovementioned confounders (Supplementary Table S1) and separately listed co-morbities per patient (Supplementary Table S2), others have not been covered. Finally, the Comparison group consisted of mixed ICU patients and has to be regarded as a heterogeneous cohort. Moreover, patients in the LuTx group had different underlying pathologies in various age groups, which might induce a certain heterogeneity within this group.

Our study has potential implication for future research in this area. Our current findings justify in-depth mechanistic studies investigating the role of PUFAs and their metabolic products during PRBC transfusion in ICU patients. Since 11 of the analytes showed accumulation in PRBCs and 22 were ubiquitously detectable in ICU patient plasma, future studies may focus on these analytes in the frame of TRIM. Of special interest may be in depth analysis of 8-HETE and/or 12-HETE as well 20-HETE, as both analytes showed an (unexpected) decrease in plasma after PRBC transfusion in ICU patients, which may only indirectly be contributed to the potential storage lesion. Furthermore, future studies may focus on non-direct mechanisms/pathways and negative feedback regulation via lipoxygenase and cytochrome P450 pathways (Dennis and Norris, 2015) or T-cell regulation (Nore et al. 2020). In addition to eicosanoids, extracellular vesicles could act as possible mediators of TRIM (Öhlinger et al. 2022). In order to improve recruitment, follow-up studies should factor in 24/7 availability of qualified study staff. In general, the prospectively selected groups of patients (i.e., Aorta and LuTx) provided reasonable case numbers and therefore seem feasible for further investigation. In addition, trauma patients may be a patient collective worth investigating, especially since these patients may be prone to complications secondary to the transfusion of PRBCs (Spinella et al. 2009; Juffermans et al., 2012).

As analyte abundances in patient plasma prior to transfusion showed only moderate fluctuation, analysis of one representative sample prior to transfusion seems justified. Hence, focus on plasma abundances within 60 minutes after transfusion may enable a more precise description of dynamics. As the co-existing diseases and other confounders reported in this study were heterogeneous, their statistical effect remains to be tested in larger, prospective studies.

In summary, recruitment of ICU patients into this transfusion study was challenging but feasible. Eicosanoid abundances increased in PRBC supernatants during storage. In plasma of ICU patients, eicosanoid abundances were ubiquitously detectable and showed limited fluctuations over time prior to transfusion. Taken together, larger clinical studies seem warranted and feasible to further investigate the role of PRBC-derived eicosanoids in TRIM.

## Data Availability

The original contributions presented in the study are included in the article/Supplementary Material, further inquiries can be directed to the corresponding author.
