# Supplementary material for: Red blood cell transfusion-related eicosanoid profiles in intensive care patients—A prospective, observational feasibility study
Source: Front Physiol. 2023 Mar 16;14:1164926. doi: 10.3389/fphys.2023.1164926 (PMC10060532; doi:10.3389/fphys.2023.1164926)
Supplement: Supplementary file 2 [file DataSheet1.pdf]

Supplementary Material

Red blood cell transfusion-related eicosanoid profiles in intensive care patients – a prospective, observational feasibility study

Pierre Raeven, Gerhard Hagn, Laura Niederstätter, Jonas Brugger, Sophia Bayer-Blauensteiner, Christoph Domenig, Konrad Hoetzenecker, Martin Posch, Gerda Leitner, Christopher Gerner, David M. Baron*

***Correspondence:**
David M. Baron (Principal investigator)
E-mail: david.baron@meduniwien.ac.at

Supplementary Methods

Eicosanoid Analysis of Human Plasma and PRBC

Samples from PRBCs and patient blood were immediately centrifuged (15 min at 726 *g* at room temperature). For protein precipitation, 500 µL of plasma was added to 2 mL of cold ethanol (abs. 99.9%, -20°C, AustroAlco) in 15 mL tubes (Corning™ Falcon™, StarLab) and stored at -20°C overnight as previously described (1). Then, concentrations of 10-100 nM of each of the internal standards (12S-HETE-d8, 15S-HETE-d8, 5-Oxo-ETE-d7, 20-HETE-d6, PGE2-d4 and 11,12-DiHETrE-d11; Cayman Europe, Tallinn, Estonia) were added to the stored precipitated samples and again stored overnight.
After overnight protein precipitation, samples were centrifuged (30 min at 4536 g and 4°C) and supernatant transferred into a new tube before ethanol was evaporated by vacuum centrifugation at 37°C until the original sample volume was restored. Eicosanoids were extracted using 30 mg mL^{-1} Strata-X solid-phase extraction columns (Phenomenex, Torrance, CA, U.S.A.). Columns were washed and equilibrated with liquid chromatography-mass spectrometry (LC-MS) grade methanol (MeOH; VWR International, Vienna, Austria) and LC-MS grade water before samples were loaded. Sample tubes were washed with 5 mL of LC-MS grade water, and eicosanoids were eluted with 500 µL methanol containing 2% formic acid (FA, Sigma-Aldrich). Solvents were evaporated under a gentle nitrogen stream at room temperature, and lipids were dissolved in 150 µL of reconstitution buffer (H_2O:ACN:MeOH + 0.2% FA - 65:31.5:3.5) including 10-100 nM of each of the internal standards (5S-HETE-d8, 8-iso-PGF2a-d4 and 14,15-DiHETrE-d11; Cayman Europe, Tallinn, Estonia).
The analytes were then separated on a Kinetex® C18 - column (Kinetex® 2.6 µm C18 100 Å, 150 x 2.1 mm (Phenomenex®)), applying a 20 min gradient flow method using a Thermo Scientific™ Vanquish™ (ultra-high performance LC). Water + 0.2% FA represented mobile phase A and ACN:MeOH (vol% 90:10) + 0.2% FA represented mobile phase B. The gradient flow profile began at 35% B, going to 90% B from 1-10 min, further increasing to 99% B from 10-10.5 min and was then held for 5 min. After decreasing to 35% B within 0.5 min, the equilibration at 35% B was performed for 4 min resulting in a 20 min total run time. The flow rate was 200 µL min^{-1}, the column temperature was 40°C and the injection volume was 20 µL. All samples were measured in technical duplicates. MS detection was performed with a Q Exactive™ HF Quadrupole-Orbitrap™ mass spectrometer (Thermo Fisher Scientific), using a heated electrospray ionization source for negative ionization. Spray voltage of 2.2 kV and a capillary temperature of 253°C were applied. Sheath gas was set to 46 arbitrary units and the auxiliary gas to 10. The scan range was set from *m/z* 250-700 with a resolution of 60,000 (at *m/z* 200) on the MS1 level and 15,000 (at *m/z* 200) on the MS2 level. Therefore, the two most abundant precursor ions were fragmented (HCD 24 normalized collision energy), preferably from an inclusion list of 31 eicosanoids and eicosanoid precursor specific *m/z* values.
Raw files generated by the Q Exactive HF Orbitrap mass spectrometer were analyzed manually with Thermo Xcalibur 4.1.31.9 (Qual browser). First, potential eicosanoids were manually identified via tandem mass spectrometry spectra using libraries from Lipid Maps depository (2) and further identification was confirmed with commercially available standards. For peak integration, the TraceFinder™ software package (version 4.1 - Thermo Scientific) was applied.
For technical reasons, abundances of 12-HETE and 8-HETE could not be analyzed separately and were therefore depicted as 12-HETE/8-HETE.

Detailed statistical analysis

To investigate if eicosanoid abundances changed over time in each of the study groups, we fitted linear mixed models with the logarithmic normalized abundance of an eicosanoid as the dependent and study group as well as the interaction of study group and time as numerical variable (0, 60, 90 or 120 minutes after start of transfusion) as the explanatory parameters. Baseline abundances were treated as if they were all obtained at T0 (as we assume no time trend before the intervention). Random intercepts and slopes were included for each patient. We tested, whether the slopes were significantly different from 0 on the log scale and whether the slopes of Aorta and LuTx significantly differed from the slope of the Comparison group. Slopes were reported with their 95% confidence intervals (CI).
In the one case where 15-HETE was under LLD in only one patient and at only one time point we imputed the only missing value by setting it to half of the smallest observable value and performed the aforementioned linear mixed model analysis.
In case of eicosanoids with at least two samples under LLD (i.e., 9-HETE and 11-HETE), we fitted tobit regression models with their logarithmic normalized abundance as the dependent variable and the interaction of time and group as the explanatory variables. Since Pat ID was included as a fixed effect,

no difference in baseline estimates between groups could be computed. No random slope for the patient was included as this would not allow for estimating slopes of the study groups. We defined the smallest observable value over the entire observation period as the lower threshold for each eicosanoid minus 5 percent respectively. Model estimates as well as lower and upper confidence bounds were transformed back to non-logarithmic normalized abundances.

Supplementary Tables

Group	Pat ID	Hours since last PRBC	Extracorporeal treatment	OP to ICU admission (days)	ICU length of stay (days)	PRBC to ICU discharge (days)	PRBC transfused in OR (units)	Salvaged blood given in OR (ml)	Total FFP in OR (units)	Total FFP in ICU (units)	Total PC in OR (units)	Total PC in ICU (units)	Blood type PRBC	Blood type patient
Aorta	34	20	-	0	3	2	2	0	0	0	0	0	A-	A-
	36	n.a.	-	0	3	1	0	800	0	0	0	0	0+	0+
	48	25	-	0	16	15	3	2153	0	0	0	0	A+	A+
	81	18	-	0	10	8	1	2800	0	0	0	0	A+	A+
LuTx	10	12	-	-7	67	64	5	500	12	28	0	9	A+	A+
	12	20	v/a ECMO	0	19	18	9	1100	11	0	3	2	A+	A+
	14	81	-	0	10	7	2	239	8	0	8	0	A+	A+
	15	12	-	0	6	3	7	250	11	0	0	0	0+	0+
	25	58	-	0	24	21	10	0	19	0	1	0	0-	AB-
	49	61	-	1	35	27	22	2600	47	0	1	0	A-	A-
	64	42	-	0	39	33	17	1580	21	0	1	0	A-	A-
	73	43	-	0	4	1	5	0	17	4	0	0	A-	A-
Comparison	17	80	-	0	21	16	7	0	7	2	0	0	0+	0+
	20	39	-	0	13	10	0	0	0	0	0	0	B+	B+
	43	58	CVVHDF	-2	22	18	0	0	0	0	0	0	AB+	AB+
	46	75	-	-26	61	34	0	0	0	0	0	0	A+	A+
	61	24	-	0	53	9	13	794	19	0	3	0	0-	0-
	68	25	-	0	2	1	3	0	0	0	0	0	A+	A+
	79	13	-	0	5	4	1	0	0	0	0	0	A+	A+
	80	n.a.	-	0	1	0	0	0	0	0	0	0	0+	0+
	87	n.a.	-	0	8	6	0	0	0	0	0	0	A+	A+

Table S1. Extended patient characteristics. Pat ID, patient identification number; PRBC, packed red blood cell; OR, operating room; ICU, intensive care unit; FFP, fresh frozen plasma; PC, platelet concentrate; LuTx, bilateral lung transplantation; v/a ECMO, veno-arterial extracorporeal membrane oxygenation; CVVHDF, continuous veno-venous hemodiafiltration.

Group	Pat ID	Co-exisiting diseases
Aorta	34	Carotid stenosis, arterial hypertension, hyperlipidemia
	36	Pulmonary emphysema, arterial hypertension, history of trans urethral resection for bladder cancer, hepatic cysts
	48	Coronary artery disease, carotic artery stenosis, history of stroke
	81	Aterial hypertension, severe mitral regurgitation
LuTx	10	Arterial hypertension, type 2 diabetes mellitus, hypertriglyceridemia
	12	Arterial hypertension, cholecystolithiasis
	14	Osteopenia
	15	None
	25	Pulmonary hypertension, insulin-dependent diabetes mellitus, osteopenia, celiac disease
	49	Insuline-dependent diabetes mellitius, osteopenia
	64	History of pneumonia and late-onset ARDS
	73	History of multiple pneumothoraces
Comparison	17	Cryptogenic cirrosis, history of multiple bariatic operations
	20	Atrial fibrillation, arterial hypertension, coronary artery disease, compensated hypothyreodism
	43	Lower arm phlegmon, type 2 diabetes mellitus, arterial hypertension, history of gastric bypass
	46	History of recent gastrectomy for gastric cancer, arterial hypertension, hyperlipidemia
	61	History of postoperative ARDS with ECMO support, renal insufficiency with history of CVVHDF
	68	Arterial hypertension, non insulin-dependent diabetes mellitus, hepatitis B-induced cirrosis
	79	History of breast cancer
	80	Dilatative cardiomyopathy,chronic renal insufficiency, hepatic steatosis, varicosis
	87	Human immunodeficiency virus infection, vertebral column fracture

Table S2. Patient co-morbidities. Pat ID, patient identification number; LuTx, bilateral lung transplantation; ARDS, acute respiratory distress syndrome; v/a ECMO, veno-arterial extracorporeal membrane oxygenation; CVVHDF, continuous veno-venous hemodiafiltration.

See attached Table_S3.xlsx

Table S3 Eicosanoid abundance (expressed as normalized area under the curve) of eicosanoids in transfused PRBCs (sheet Data_table_PRBC) and in patient plasma (Data_table_plasma) at predefined time points (T-2, T-1, T0, T1, T2, T3). Sample T2 of patient 81 was lost and not analyzed. Pat ID; patient identification number; LuTx, bilateral lung transplantation; PRBC, pack red blood cells; AA, arachidonic acid; HETE, hydroxyeicosatetraenoic acid; EET, epoxyeicosatrienoic acid; DiHETrE, dihydroxyeicosatrienoic acid; HpODE, hydroperoxyoctadecadienoic acid; HODE, hydroxyoctadecadienoic acid; EpOME, epoxyoctadecenoic acid; DiHOME, dihydroxyoctadecenoic acid; HOTrE, hydroxyoctadecatrienoic acid; HEPE, hydroxyeicosapentaenoic acid; HDoHE, hydroxydocosahexaenoic acid; <LLD, under lower limit of detection; n.s., no sample.

	5-HETE	9-HETE	11-HETE	12-HETE/8-HETE	15-HETE	20-HETE	AA
**Baseline abundance Comparison**	0.20 [0.13, 0.31]	0.46 [0.33, 0.63]	0.52 [0.41, 0.65]	0.59 [0.35, 0.98]	0.29 [0.17, 0.49]	0.68 [0.42, 1.11]	33.20 [22.86, 48.21]
**Relative mean difference at baseline Aorta**	1.24 [0.57, 2.72]	N/A	N/A	0.97 [0.39, 2.42]	1.16 [0.45, 2.98]	1.03 [0.43, 2.48]	0.61 [0.31, 1.20]
**Relative mean difference at baseline LuTx**	1.42 [0.75, 2.68]	N/A	N/A	0.61 [0.29, 1.28]	1.01 [0.47, 2.17]	0.70 [0.34, 1.42]	0.58 [0.34, 1.00]
**Slope Comparison**	0.99 [0.86, 1.14]	1.08 [0.94, 1.24]	0.99 [0.82, 1.19]	0.86 [0.78, 0.95]**	0.99 [0.91, 1.07]	0.88 [0.79, 0.97]**	0.92 [0.84, 1.02]
**Slope Aorta**	1.21 [0.98, 1.49]	1.02 [0.82, 1.28]	1.14 [0.88, 1.48]	0.88 [0.75, 1.03]	0.96 [0.85, 1.09]	1.02 [0.88, 1.18]	1.01 [0.88, 1.17]
**Slope LuTx**	1.01 [0.87, 1.17]	0.91 [0.78, 1.06]	1.10 [0.97, 1.25]	0.93 [0.84, 1.04]	0.98 [0.90, 1.07]	0.85 [0.77, 0.94]**	0.96 [0.87, 1.06]
**Slope total cohort**	1.07 [0.97, 1.18]	1.00 [0.91, 1.11]	1.07 [0.96, 1.21]	0.89 [0.83, 0.96]**	0.98 [0.92, 1.03]	0.91 [0.85, 0.98]**	0.97 [0.91, 1.03]
**Relative change slope Aorta**	1.22 [0.94, 1.57]	0.95 [0.73, 1.23]	1.16 [0.84, 1.59]	1.03 [0.85, 1.24]	0.97 [0.84, 1.13]	1.17 [0.98, 1.39]	1.09 [0.92, 1.30]
**Relative change slope LuTx**	1.02 [0.83, 1.25]	0.84 [0.68, 1.04]	1.12 [0.89, 1.40]	1.09 [0.94, 1.26]	0.99 [0.88, 1.12]	0.97 [0.84, 1.12]	1.04 [0.91, 1.19]

Table S4 Values in the table correspond to the estimates of the linear mixed model and their 95% confidence intervals. “Baseline abundance” corresponds to the estimated average eicosanoid abundances in the Comparison group at baseline (60 to 0 minutes before transfusion, T-3 to T0. “Relative mean difference at baseline” corresponds to the estimated relative differences of eicosanoid abundances in Aorta and LuTx with regards to the Comparison group at baseline. Dynamics of eicosanoid abundances over time (baseline to 120 minutes after start of transfusion) are displayed as estimated slopes in the study groups. The respective estimated relative change of the slopes in the Aorta and the LuTx relative to the Comparison group can be found under “Relative change slope”. HETE, hydroxyeicosatetraenoic acid; AA, arachidonic acid; LuTx, bilateral lung transplantation **p<0.01; N/A, not applicable (n too low).

Supplementary Figures

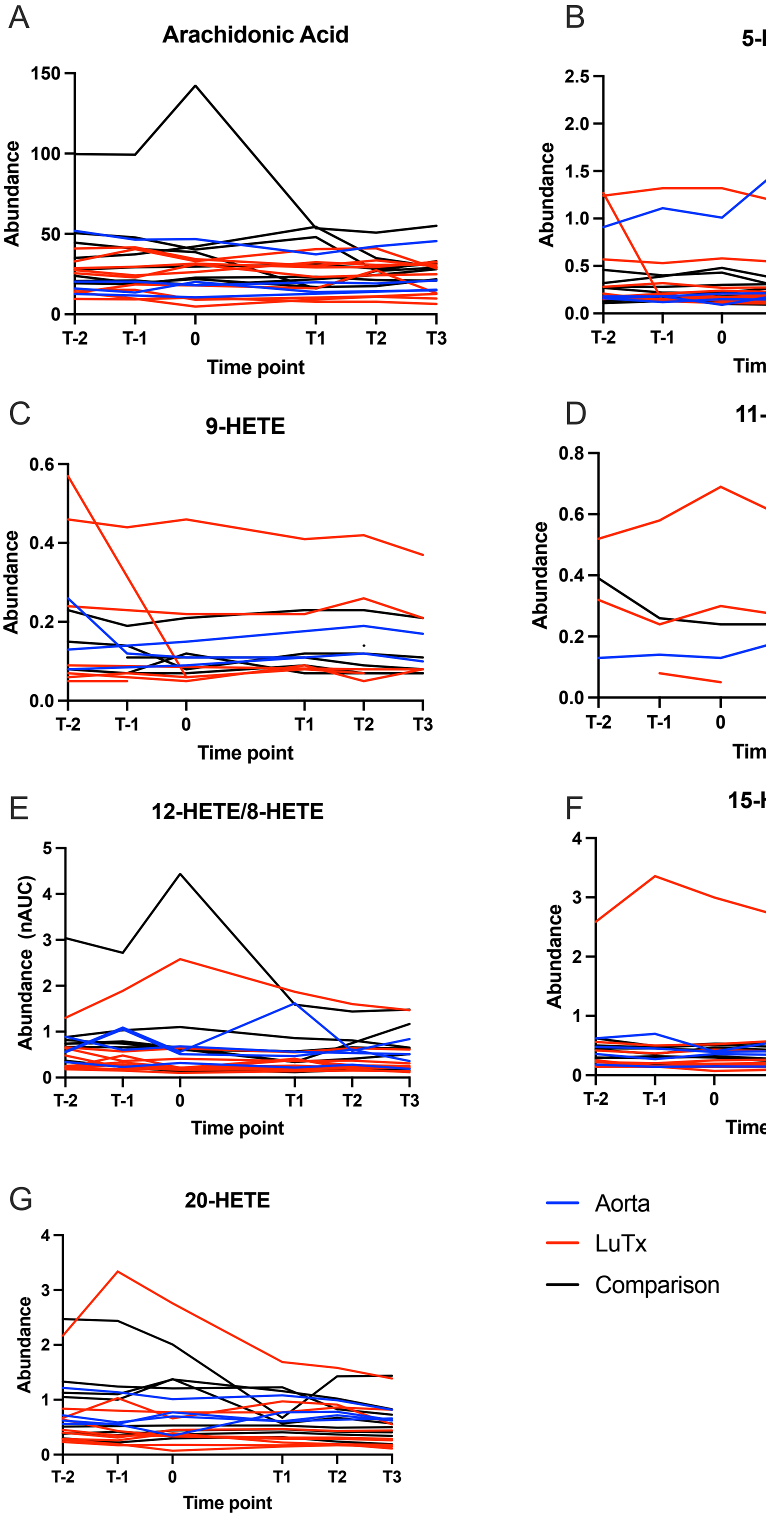

Figure S1 Normalized area under the curve values for predefined eicosanoids in patient plasma at predefined time points (T-2, T-1, T0, T1, T2, T3) and in transfused PRBCs. A) Arachidonic Acid, B) 5-HETE, C) 9-HETE, D) 11-HETE, E) 12-HETE/8-HETE, F) 15-HETE, G) 20-HETE. Lines depict dynamics of individual patients by group: Aorta (blue lines), LuTx (red lines), Comparison (black lines). HETE, hydroxyeicosatetraenoic acid; LuTx, bilateral lung transplantation.

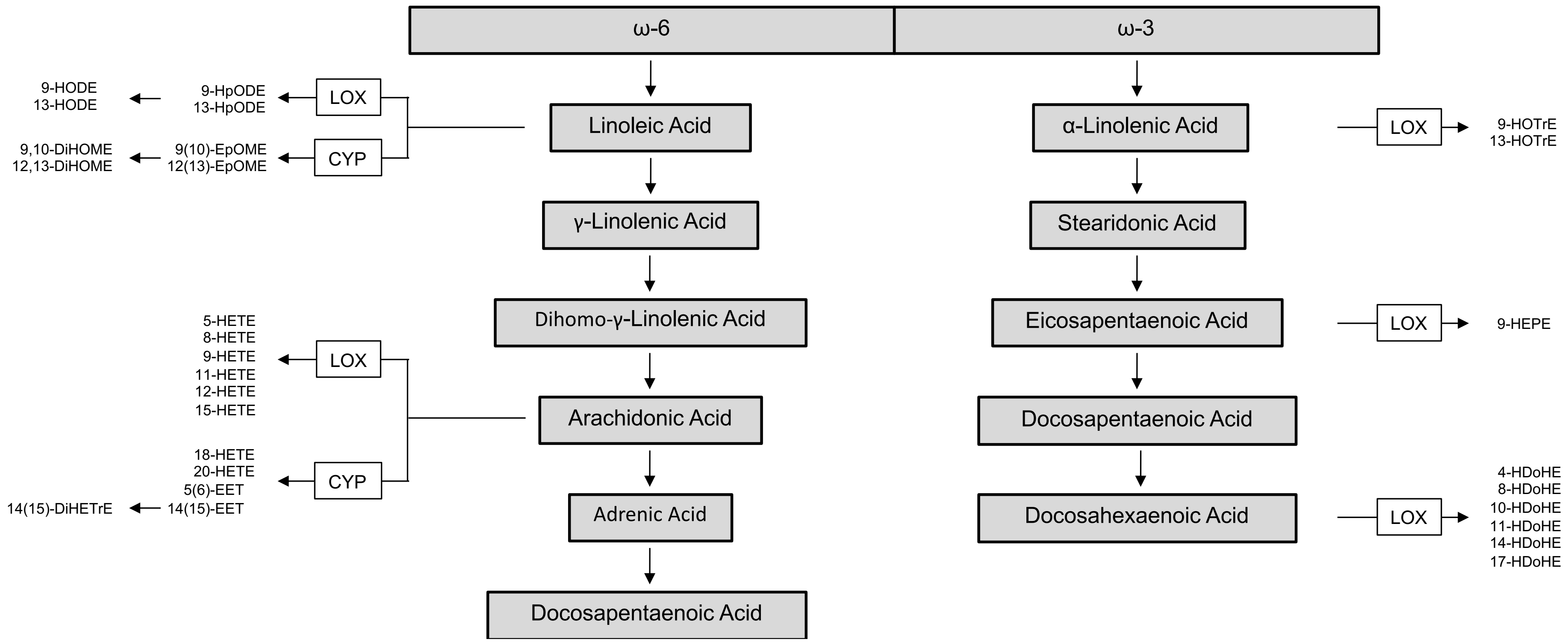

Figure S2. Metabolic pathways related to analytes assessed. Grey boxes depict Omega-6 and Omega-3 poly-unsaturated fatty acid precursors. White boxes depict enzymatic pathways. AA, arachidonic acid; HETE, hydroxyeicosatetraenoic acid; EET, epoxyeicosatrienoic acid; DiHETrE, dihydroxyeicosatrienoic acid; HpODE, hydroperoxyoctadecadienoic acid; HODE, hydroxyoctadecadienoic acid; EpOME, epoxyoctadecenoic acid; DiHOME, dihydroxyoctadecenoic acid; HOTrE, hydroxyoctadecatrienoic acid; HEPE, hydroxyeicosapentaenoic acid; HDoHE, hydroxydocosahexaenoic acid. Based on (3, 4).

References

1. Leitner GC, Hagn G, Niederstaetter L, Bileck A, Plessl-Walder K, Horvath M, et al. INTERCEPT Pathogen Reduction in Platelet Concentrates, in Contrast to Gamma Irradiation, Induces the Formation of trans-Arachidonic Acids and Affects Eicosanoid Release during Storage. Biomolecules. 2022;12(9).
2. Fahy E, Sud M, Cotter D, Subramaniam S. LIPID MAPS online tools for lipid research. Nucleic Acids Res. 2007;35(Web Server issue):W606-12.
3. Gabbs M, Leng S, Devassy JG, Monirujjaman M, Aukema HM. Advances in Our Understanding of Oxylipins Derived from Dietary PUFAs. Adv Nutr. 2015;6(5):513-40.
4. Steer CD, Lattka E, Koletzko B, Golding J, Hibbeln JR. Maternal fatty acids in pregnancy, FADS polymorphisms, and child intelligence quotient at 8 y of age. Am J Clin Nutr. 2013;98(6):1575-82.
